# Characterization of the chicken inward rectifier K^+ ^channel *IRK1/Kir2.1 *gene

**DOI:** 10.1186/1471-2164-5-90

**Published:** 2004-11-29

**Authors:** Hideki Mutai, Lawrence C Kenyon, Emily Locke, Nami Kikuchi, John Carl Oberholtzer

**Affiliations:** 1 Department of Pathology and Laboratory Medicine, Division of Neuropathology, University of Pennsylvania School of Medicine, Philadelphia, PA, USA; 2 Present address: Department of Otolaryngology, Program of Neuroscience, Harvard Medical School and Massachusetts Eye and Ear Infirmary, Boston, MA, USA; 3 Present address: Department of Pathology, Anatomy, and Cell Biology, Thomas Jefferson University, Philadelphia, PA, USA

## Abstract

**Background:**

Inward rectifier potassium channels (IRK) contribute to the normal function of skeletal and cardiac muscle cells. The chick inward rectifier K^+ ^channel cIRK1/Kir2.1 is expressed in skeletal muscle, heart, brain, but not in liver; a distribution similar but not identical to that of mouse Kir2.1. We set out to explore regulatory domains of the *cIRK1 *promoter that enhance or inhibit expression of the gene in different cell types.

**Results:**

We cloned and characterized the 5'-flanking region of *cIRK1*. *cIRK1 *contains two exons with splice sites in the 5'-untranslated region, a structure similar to mouse and human orthologs. *cIRK1 *has multiple transcription initiation sites, a feature also seen in mouse. However, while the chicken and mouse promoter regions share many regulatory motifs, *cIRK1 *possesses a GC-richer promoter and a putative TATA box, which appears to positively regulate gene expression. We report here the identification of several candidate cell/tissue specific *cIRK1 *regulatory domains by comparing promoter activities in expressing (Qm7) and non-expressing (DF1) cells using *in vitro *transcription assays.

**Conclusion:**

While multiple transcription initiation sites and the combinatorial function of several domains in activating *cIRK1 *expression are similar to those seen in *mKir2.1*, the *cIRK1 *promoter differs by the presence of a putative TATA box. In addition, several domains that regulate the gene's expression differentially in muscle (Qm7) and fibroblast cells (DF1) were identified. These results provide fundamental data to analyze *cIRK1 *transcriptional mechanisms. The control elements identified here may provide clues to the tissue-specific expression of this K^+ ^channel.

## Background

The inward rectifier potassium channel IRK1/Kir2.1 helps controls cell excitability through setting the resting membrane potential [1]. Its dominant role of inward rectification for the normal function of skeletal and cardiac muscles is shown by the complete loss of inward rectifying current and K^+^-induced dilations in arterial myocytes from Kir2.1 knockout mice [2] and periodic paralysis, and by cardiac arrhythmias in Anderson's syndrome caused by point mutation of human Kir2.1 [3].

Kir2.1 expression is detected in excitable cells in brain, heart, and skeletal muscle in both mouse and chick [4-8]. In addition, chicken IRK1 (cIRK1) is expressed in the cochlea [8], a feature not observed in mammals [9,10]. In this report, we first analyzed *cIRK1 *genomic DNA to identify transcriptional initiation sites and distinct motifs that are important for the expression of this potassium channel gene. Using *in vitro *promoter assays with fragments of the cloned *cIRK1 *locus, we also identified several candidate control domains that may participate in regulating the channel's exquisite tissue-specific transcription.

## Results

### Structure of the chick IRK1 genomic locus

**Figure 1:**
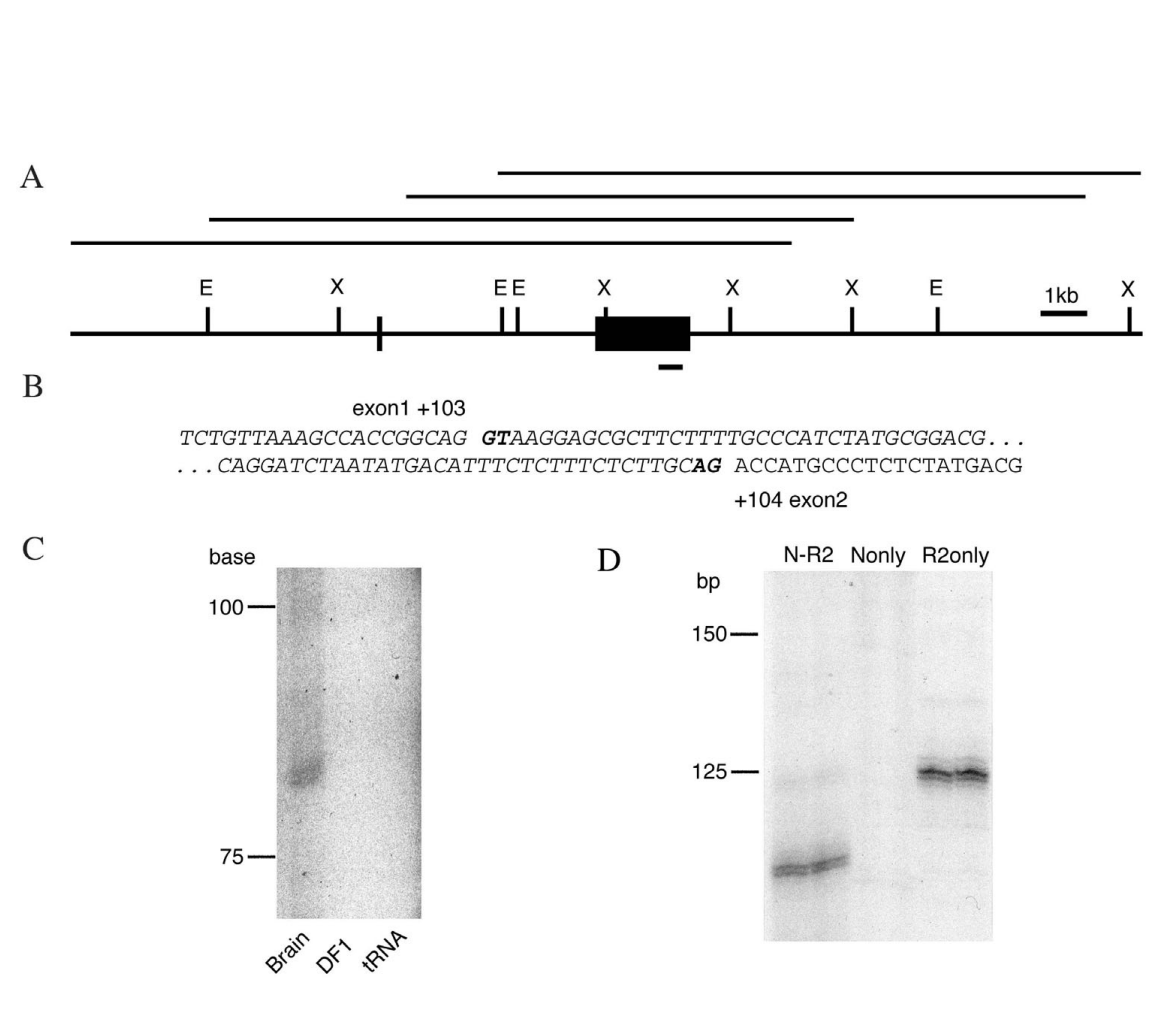
**Genomic structure of *chick IRK1 *and determination of transcription initiation sites **(A) Restriction map of chick *IRK1/Kir2.1 *genomic locus. The four overlapping genomic clones are shown above the restriction map. Solid boxes indicate exons. E; EcoRI site, X; XbaI site. The probe used for northern blots is underlined. (B) Genomic sequence surrounding splice sites. Exon 1 ends at +103 and exon 2 begins at +104 (based on *cIRK1 *cDNA, U20216), as indicated. Intronic sequences are in italics; bold letters indicate donor site GT and acceptor site AG. (C) Primer extension analysis. Approximate size, in bases, is indicated on the left. Products of about 80 bases were present in brain, but not in DF1 fibroblasts or in controls (tRNA). (D) Determination of transcription initiation sites using 5'RACE. Shown are the results of the secondary PCR using the 5'-nested primer and a primer designed at 64-41 of *cIRK1 *cDNA (N-R2), and controls using the 5'-nested primer only (N only) or the reverse primer only (R2 only).

We began this study by isolating *cIRK1 *genomic clones from a chicken genomic DNA phage library. A series of overlapping clones were isolated by screening the library using full length *cIRK1 *cDNA as a specific probe. Approximately 6.5 kilobase pairs (kb) of the *cIRK1 *5'-flanking region were sequenced (Genbank AF375660), including exon 1 and a portion of the intron. *cIRK1 *contains two exons: exon 1 includes only upstream non-coding sequence (5'-untranslated region, 5'UTR) while exon 2 includes 5'UTR (216 bp), the full open reading frame (1,284 bp), and the 3'UTR (520 bp) (Figure 1A). The single intron is estimated to be approximately 4.9 kb in length. A comparison of the cDNA and genomic sequences shows the splice site to be located between positions 103 and 104 of *cIRK1 *cDNA (GenBank U20216). Sequence data also showed that the intron had consensus donor (GU) and acceptor (AG) sequences (Fig. 1B). This genomic structure of the *cIRK1 *locus resembles that of *mKir2.1 *[11], which possesses two exons separated by a 5.5 kb intron. In a previous report [8], an approximately 5.5 kb *cIRK1 *transcript was detected, in addition to one 2.5 kb in length, in brain, cerebellum, heart, skeletal muscle, and cochlea. Since we have identified polyadenylation signals at bp positions 1,645–1,650 and 1,865–1,870 of the cDNA, we conclude that *cIRK1 *has 2 exons and no additional exon in the 3'UTR. This is also supported by the fact that multiple attempts to extend the cDNA by library screening or 3'RACE were not successful.

### Identification of transcription initiation sites

**Figure 2:**
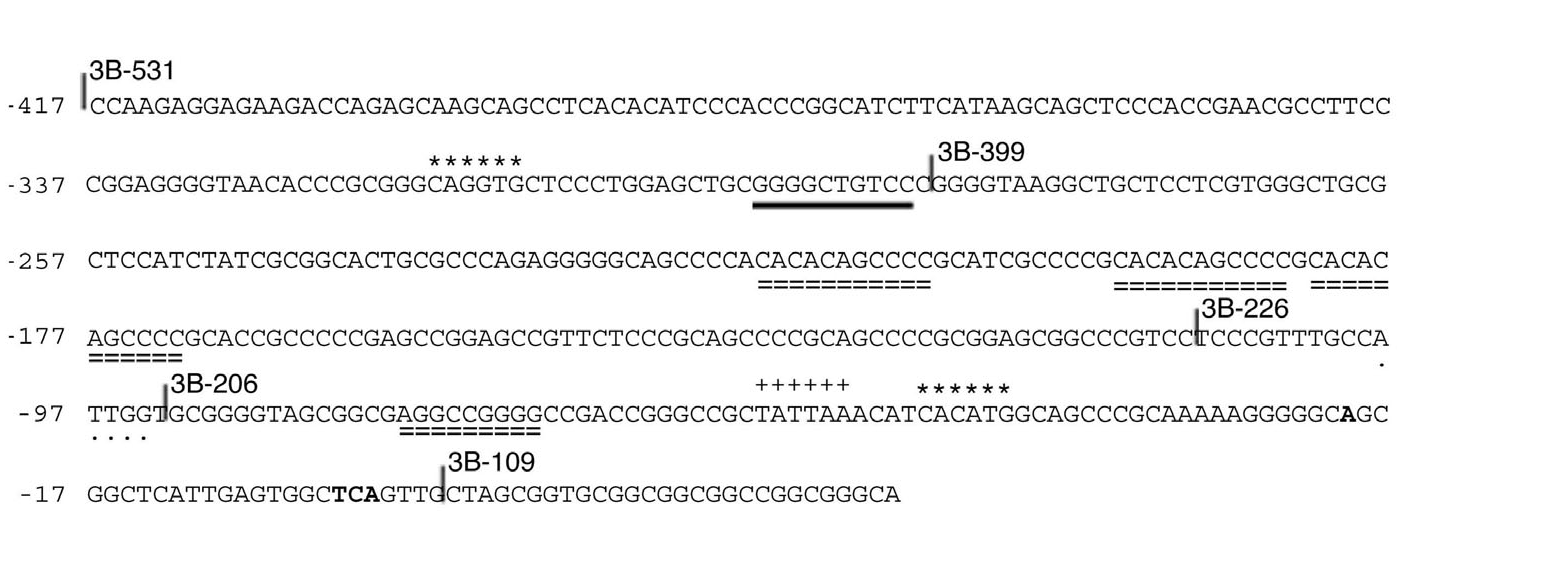
**Motifs in 5'-flanking region of *cIRK1 ***DNA sequence of the 5'-flanking region (from -417 to +33) is shown. Highlighted are the positions of two E boxes (asterisks), a NF-κB site (heavy underline), a putative TATA box (plus signs), four Sp1 sites (dashed double underlines), and an NF-Y binding site (dotted underline). Transcription initiation sites are shown in bold, with the most downstream site numbered +1. The 5'-ends of the fragments used in the promoter assays are shown with slashes and the construct names (see also Figure 4).

**Figure 3:**
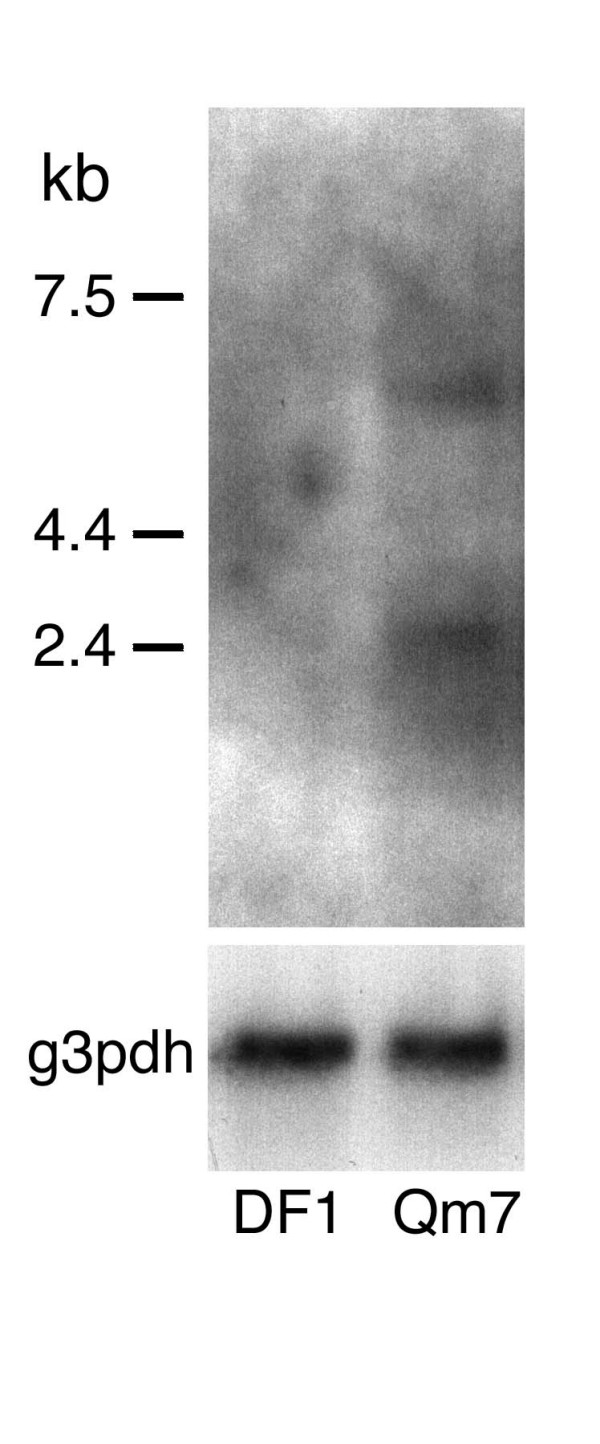
***IRK1 *is expressed in Qm7 cells, but not in DF1 cells. **Northern blot analyses were performed using 5 μg of poly A(+) RNA from each cultured cell type. Two bands whose sizes (5.5 kb and 2.5 kb) are similar to those previously reported in chick tissues [8] were detected in Qm7 cells but not in DF1 cells. g3pdh is included as a loading control.

Before studying the motifs and elements of the promoter region, we sought to determine the transcription initiation sites of *cIRK1 *using primer extension and 5'RACE. When the specific reverse complementary sequence to the *cIRK1 *5'UTR at positions 61–41 was utilized as a primer for reverse transcription, a product of approximately 80 bases was generated from brain mRNA (Fig. 1C). Specificity of cDNA synthesis was determined by the absence of the band from yeast tRNA and DF1 cells – a chicken embryonic fibroblast cell line which does not express detectable levels of cIRK1 mRNA (Fig. 3). To confirm the precise transcriptional initiation sites, oligo-capped RNA based 5'RACE [12] was carried out using brain total RNA as template. This method results in RNA of which only transcripts with intact 5'-ends are capped by an RNA-oligo. We then reverse-transcribed this RNA using a specific primer R1 designed at positions 146–122 of *cIRK1 *cDNA. PCR was first performed with the supplied (GeneRacer) 5'-primer and the reverse primer R1. Subsequent nested PCR, using the supplied 5'-nested primer and a second reverse primer R2 designed at positions 64–41 of *cIRK1 *cDNA, produced multiple fragments. Those of approximately 110 bp were determined to be specific products of the primer set (N-R2) because they were not detected when the PCR was carried out using either the 5'-nested primer alone (N only) or the R2 primer alone (R2 only, Fig. 1D). The PCR fragments were cloned and multiple clones were sequenced. All of them contained 30 bp of 5'-capped oligo plus several lengths of *cIRK1 *transcript (80, 81, 82, and 100 bp), which occurred with similar frequencies. Therefore, the actual transcribed 5'-flanking regions were 16, 17, 18, or 36 nucleotides longer than the 5'-end of the clone originally isolated from a chick cochlea cDNA library [8]. The confirmed initiation sites are shown in bold in Fig. 2. The 3'-most initiation site, located 16 nucleotides upstream of the 5'-end of the previously reported *cIRK1 *cDNA, was numbered +1. Our identification of multiple transcription initiation sites indicates that exon 1 of *cIRK1 *can be 119, 120, 121, or 139 bases in length.

### Comparison of the 5'-flanking regions of *cIRK1 *and *mKir2.1*

We then analyzed the motifs contained in the *cIRK1 *5'-flanking region, based on the presumption that there might be significant divergence between chicken and mammal *IRK1/Kir2.1 *promoter regions that could account for their different gene expression patterns. The 5'-flanking region of *cIRK1*, shown in Fig. 2, shares no evident homology with the comparable region of the *mKir2.1 *promoter [11]. The corresponding region of the putative human *Kir2.1 *promoter shows high similarity to the *mKir2.1 *promoter (62.6% over1,017 bp), however, the *hKir2.1 *promoter also failed to show significant homology with the *cIRK1 *5'-flanking region. While both the *cIRK1 *5'-flanking region and the *mKir2.1 *promoter have high GC contents, the chick promoter is substantially more GC-rich (71.0% in chick and 62.8% in mouse from position -390 to -1) and contains 38 CpG dinucleotides as compared to 17 CpGs in mouse. The *cIRK1 *promoter region has four consensus Sp1 binding elements (at positions -216, -194, -182, and -70; Fig. 2), while the *mKir2.1 *promoter contains three Sp1 sites [11]. Consensus binding sites for several other factors were also identified. E boxes (CANNTG) were present at -316 and at -46, and an inverted CCAAT (NF-Y element) [13] was found at -98, while the *mKir2.1 *promoter region contains 3 E boxes and one NF-Y element. A striking difference from *mKir2.1 *is that the *cIRK1 *promoter was found to contain a putative TATA box (TATTAA), absent in *mKir2.1*, at -56. Overall, while GC-rich domains and motifs are present in both the chick and mouse promoter regions, the extent and number of the motifs are different between the *cIRK1 *and *mKir2.1 *promoters. In addition, a TATA box-like sequence is present only in the chick promoter.

### *In vitro *promoter analysis and cell-specific regulatory regions

**Figure 4:**
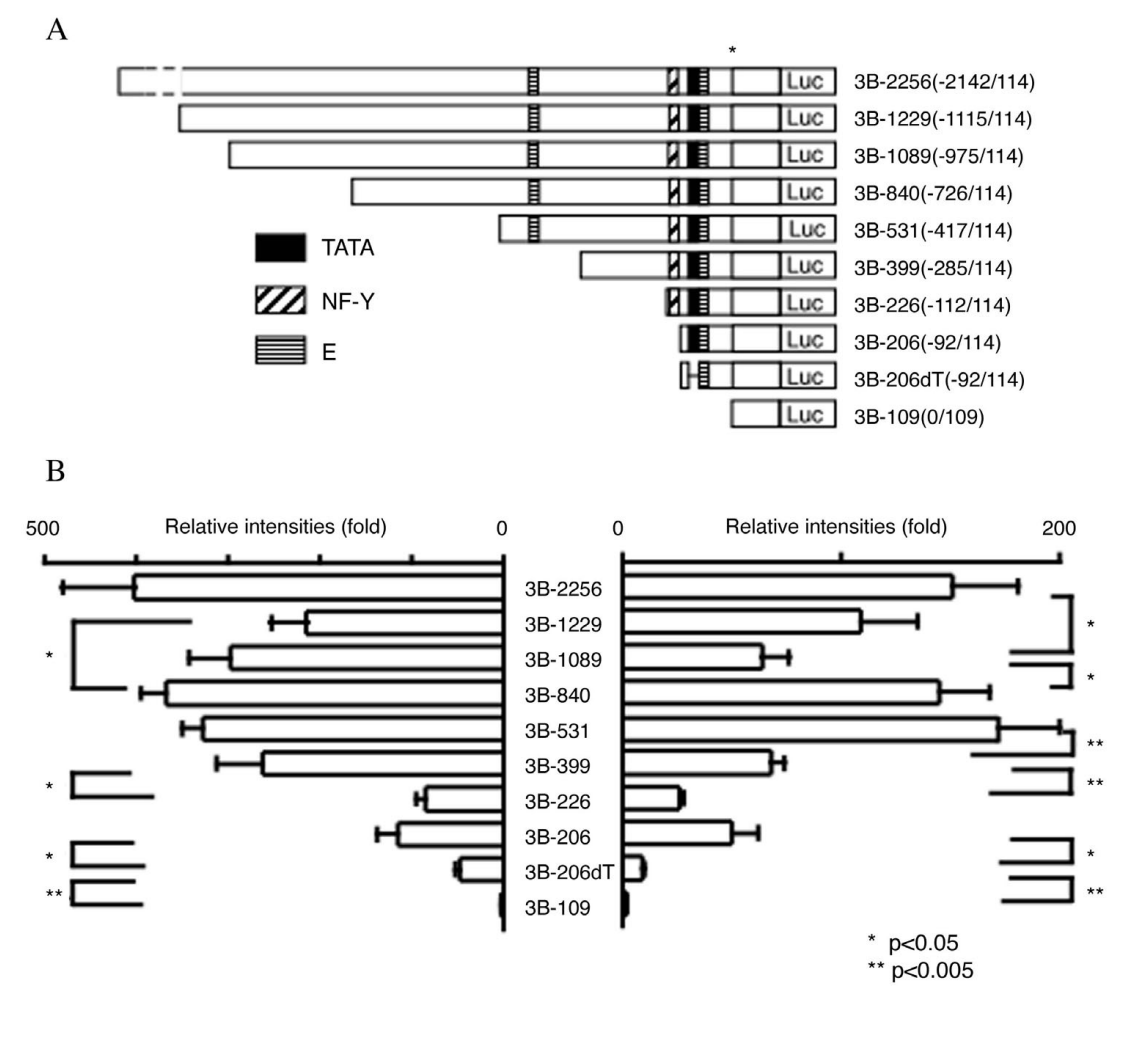
**Identification of domains that affect cIRK1 expression **(A) Constructs used in *in vitro *promoter assays. The putative TATA box, an NF-Y binding site, and an E box are shown in black, hatched and lined boxes, respectively. Numbers in parentheses indicate the length in bp of the promoter region/exon 1 included in each test plasmid. The transcription initiation site is marked with an asterisk. Luc, luciferase reporter gene. (B) *In vitro *transcription analysis. Plasmid constructs were transiently transfected into DF1 (left) or Qm7 cells (right). Results are shown as fold increases compared with the basal luciferase activity from cells transfected with control pGL-3B vector. Standard errors are shown as bars. *; p value < 0.05, **; p < 0.005.

We next sought to determine which of these avian-specific upstream elements, including the putative TATA-box, might play a role in *cIRK1 *transcription. First, we tested whether the 5'-flanking region up to approximately 2 kb contained sufficient elements to activate *cIRK1 *transcription, and then we investigated the minimal promoter region by constructing a series of 5'-flanking region deletion fragments, including a portion of exon 1, inserted into plasmids upstream of the luciferase reporter (Fig. 4A). Promoter activities were measured following transient transfection into DF1 or quail myoblast Qm7 cells, which do not and do express endogenous avian IRK1, respectively (Fig. 3). We hypothesized that identification of tissue specific regulatory regions of *cIRK1 *could be achieved by a comparison of reporter gene expression data obtained from these two cell lines. Figure 4B shows that *cIRK1 *transcription was activated in many of the deletion constructs in both DF1 and Qm7 cells, indicating that both contain sufficient factors to activate cIRK1 expression, whether or not they express the gene endogenously. However, promoter activities were differentially regulated in the two cell types when the relatively long 5'-flanking regions were included in the constructs upstream of the reporter luciferase gene (Fig. 4B). First, in Qm7 cells, deletion of a domain from position -2142 to -976 resulted in an approximately 60% loss of promoter activity (3B-2256 versus 3B-1089). In addition, deletion of the segment from -975 to -727 resulted in a recovery of activity (3B-840). This indicates that the region between -975 and -727 can function to repress transcription, but that this repression can be overcome by upstream elements. These effects were generally not seen in DF1 cells, except that 3B-840 exhibited somewhat higher activity than did 3B-1229. The region between -1115 and -976 showed a weak inhibitory effect on reporter expression in DF1 cells, while it had a positive regulatory function in Qm7 cells. Finally, a dramatic loss (59%) of promoter activity was observed upon deletion of the domain from -417 to -286 in Qm7 cells (3B-531 versus 3B-399). While this effect was substantial in Qm7 cells, it was absent in DF1 cells. These data indicate that the element(s) in this region activate IRK1 gene expression only in the myoblast cell line Qm7.

The relative promoter activities exhibited by 3B-399 and shorter constructs were similar in the two cell lines. Activities decreased significantly between 3B-399 and 3B-226, but not between 3B-226 and 3B-206. Candidate active regulatory elements in this region included three Sp1 consensus sites arranged in tandem at -216, -194, and -182. The construct 3B-206, which lacks the NF-Y element activated transcription as well or slightly better than 3B-226. Point mutation of this element in the *mKir2.1 *minimal promoter resulted in a significant increase of promoter activity, suggesting a significant role for the NF-Y element in mouse compared with chicken [11]. Specific mutation of the putative TATA box led to a 60.3 % loss of activity in DF1 cells, and an 80.6 % loss in Qm7 cells, but not to a complete loss of promoter activity. The construct 3B-109 showed no significant activity compared with the mock vector, indicating that the 5'-flanking region up to -92 upstream is necessary for promoter activity. Taken together, two distal regions, -1115 to -727 and -417 to -286, were found to be candidate cell/tissue specific regulatory domains in the *cIRK1 *promoter, while the 5' flanking region proximal to -285 is predicted to activate ubiquitously. The GC rich regions, including Sp1 motifs, and the putative TATA box regulate the promoter positively.

## Discussion

This is the first report describing a promoter analysis of an avian potassium channel. Our data show that *cIRK1 *and *mKir2.1 *have similar genomic organizations comprised of a single intron dividing the 5'UTR. In addition, in the 5'-flanking region, the *cIRK1 *promoter appears to contain a unique putative TATA box. Our results demonstrating positive regulatory activity of the domain that includes the TATTAA sequence strongly suggest that this element does indeed recruit and bind TATA-binding protein [14], thereby contributing significantly to gene expression. No TATA boxes are found in mammalian K^+ ^channel promoters such as those for *Kir2.1 *[9], *Kir3.1 *[15], *Kir3.4 *[16], *Kv1.4 *[17], *Kv1.5 *[18] and *Kv3.1 *[19,20]. Further studies may determine whether chick orthologous K^+ ^channel genes share their genomic organizations with those of mammals.

It should also be emphasized that elements other than the putative TATA box also participate in, although to a lesser extent, transcription initiation of *cIRK1*. Just upstream of the transcription initiation site lie several binding sites for transcriptional regulation, including Sp1 sites and an E box; these are also present in *mKir2.1 *and have been shown to activate that gene's expression [11]. While the significant role shown for the NF-Y site in *mKir2.1 *[9] was not apparent in the case of *cIRK1 *promoter activation, Sp1 and E box-binding proteins may work cooperatively in promoter activation, as this mechanism has been reported in the study of telomerase reverse transcriptase gene regulation [21]. Overall, the difference in transcriptional regulation of *IRK1/Kir2.1 *between chicken and mouse is that regulation of *cIRK1 *expression depends not only on the Sp1 sites and E box, but also on the putative TATA box. It is known that *IRK1/Kir2.1 *is expressed in the sensory epithelium of the chick cochlea [8], but that it is not expressed in the mature cochlea of the mouse [9] or rat [10]. Whether the putative TATA box leads to the expression of *cIRK1 *in the cochlea awaits further analysis.

While the results of our *in vitro *transcription analyses indicate that *cIRK1 *transcription can be activated both in expressing Qm7 and in non-expressing DF1 cell lines, and therefore likely in a variety of tissues, they also showed the existence of several cell specific regulatory domains. Examination of transcriptional regulation by the region -417 to -286 indicated that element(s) in this region increase promoter activity only in the myoblast cell line Qm7. Candidate elements within this region that might have cell-specific activity are an E box (CAGGTG) at -316 and an activating component of transcription activator NF-κB (GGGRNNYYCC; where R is A or G, Y is C or T, and N is any base) at -296 [22,23]. Various mouse helix-loop helix transcription factors such as atonal homolog Math1 [24] and MyoD [25] are reported to associate with E boxes and are required for tissue-specific cell type determination. The chicken Math1 ortholog Cath1 is expressed in the developing brain regions that express cIRK1 [26]. However, our co-expression of Math1 with the *cIRK1 *promoter construct 3B-531 did not enhance promoter activity (data not shown).

We also identified a region between -1115 and -727 with weak repressor activity that exhibited different properties in the two cell lines. Potential binding motifs present in this region are: a site for interferon regulatory factor-1 at -1030, an AP-4 site at -978, a thyroid hormone beta receptor binding element (TRE) at -962, another E box at -954, a binding site for the potent repressor CTF/NF-I at -859 [29], and a *Drosophila *homeotic gene *Antennapedia *binding motif [30] at -786. Among these, TRE has been shown to regulate a fast-activating K^+ ^conductance in inner hair cells [27], and an E box has been shown to function as a repressive element when bound by Mad1/Max in differentiated HL60 cells [28].

Our observation of the transcriptional activation of *cIRK1 *in DF1 cells which do not express the gene endogenously clearly suggests the existence of additional mechanisms to repress *cIRK1 *in DF1 cells. Since domains acting to strongly silence *cIRK1 *expression in DF1 cells were missing in the 5'-flanking region characterized in this study, additional distant control regions may lie further upstream, downstream, or in the intronic sequences. Although at this point clearly speculative, the existence of many CpG dinucleotides in *cIRK1 *suggests the possibility of gene repression through DNA methylation of CpG dinucleotides leading to heterochromatin condensation of the adjacent genomic locus [31]. Tissue-specific expression of cIRK1 might consequently be explained by a combination of regulation via the specific motifs studied here, regulation via additional domains outside this region, and the potential modification of the *cIRK1 *genomic locus through DNA methylation.

## Conclusions

We have identified multiple transcription initiation sites and several candidate regulatory elements in the chicken potassium channel gene *cIRK1*. These results provide fundamental data to further analyze *cIRK1 *transcriptional mechanisms. While the use of multiple transcription initiation sites and the combinatorial participation of multiple domains in activating *cIRK1 *expression are similar to those seen for mouse *Kir2.1*, the *cIRK1 *promoter is distinct in that it exhibits a higher GC-content than does the *mKir2.1 *promoter, and by the presence of a functional putative TATA box that is not observed in the *mKir2*.1 promoter. Transcriptional control domains identified here form the foundation of an in-depth analysis of tissue-specific expression of this K^+ ^channel as well as the species-specific expression of cIRK1 in the chicken cochlea.

## Methods

### cIRK1 genomic cloning and upstream sequence analysis

Chick *IRK1 *genomic clones were isolated by screening a chick genomic library in the lambda FIX II vector (Stratagene) using *cIRK1 *cDNA (GenBank U20216) as a screening probe. Briefly, the probe was random primer-labeled with ^32^P-α-dCTP and hybridized with phage plaques blotted onto HybondN nylon membranes (Amersham Biosciences) overnight at 65°C in 6x SSC, 250 μg/ml salmon sperm DNA, 5x Denhardt's solution, and 0.1% SDS. The membranes were washed in 1x SSC and 0.1% SDS, and exposed to x-ray film. Four positive clones were further analyzed following digestion with XbaI and/or EcoRI, and subcloning into pBlueScript-KS. The promoter region (AF375660) was sequenced from both ends. Comparisons between the putative human *Kir2.1 *promoter region (AC005242), the mouse *Kir2.1 *promoter region (AF072673), and the coding regions of *mKir2.1 *cDNA (AF021136) and *hKir2.1 *cDNA (U24055) were performed using the Wilbur-Lipman DNA alignment method. Transcription factor binding elements were predicted based on the TRANSFAC algorithm [32] and the transcription element search system .

### Primer extension, 5'RACE, and northern blot analysis

Total RNA was extracted from tissues and cells using TRIZOL reagent (Invitrogen) and treated with DNaseI. Three micrograms of total RNA (brain, DF1, or yeast tRNA) were reverse transcribed with ^32^P-end-labeled primer in 10 mM DTT, 50 μg/ml actinomycin D and 0.5 mM dNTP using Superscript II (Invitrogen) at 47°C for 60 min. The primer was designed at 61–41 of *cIRK1 *cDNA (U20216) [5'-TGT TAA GAT CCG CGG GGA CAC-3']. Reaction mixtures were fractionated on 7% polyacrylamide (PAA)/7 M Urea gels. The 3'-most transcription initiation site was numbered +1.

RNA ligase-mediated rapid amplification of 5' cDNA ends (5'RACE) was carried out using the GeneRacer kit (Invitrogen). In brief, 3 μg of total RNA were dephosphorylated, decapped and ligated with a 44-base RNA-oligo, according to manufacturer protocols. Next, the RNA was reverse transcribed using R1 primer designed at 146-122 of *cIRK1 *cDNA [5'-GCA GAG TTA GCT TAA CAA GTA ACC G-3'] at 42°C for 1 hr. The PCR was performed in a reaction mix containing 1x PCR buffer, 200 nM each of 5'-forward primer [5'-CGA CTG GAG CAC GAG GAC ACT GA-3'] and R1, 100 μM dNTPs, 5 μCi ^32^P-α-dCTP, 5% DMSO, and 1.25 U of AmpliTaq (Perkin-Elmer). Reaction conditions were; 94°C for 3 min, 5 cycles at 94°C for 1 min, 57°C for 5 min, 72°C for 2 min, and then 30 cycles at 94°C for 1 min, 57°C for 1 min, 72°C for 1 min, followed by 72°C for 5 min. Nested PCR was performed using the same conditions, with first round PCR products as template, and using the 5'-nested forward primer [5'-GGA CAC TGA CAT GGA CTG AAG GAG TA-3'] and R2 primer designed at 64-41 of *cIRK1 *cDNA [5'-GGG TGT TAA GAT CCG CGG GGA CAC-3']. Reaction products were eluted from the 7% non-denaturing PAA gel, re-amplified, then ligated into the pCR4-TOPO-TA cloning vector (Invitrogen) and sequenced.

Northern blotting was performed using poly A(+) RNA isolated using the FastTrak 2.0 kit (Invitrogen). Five micrograms of poly A(+) RNA were applied to each lane, fractionated on a 0.8% agarose gel, and transferred to a HybondN membrane. The membrane was hybridized with a random-primed ^32^P-labeled *cIRK1 *cDNA fragment (522 bp; 1,384–1,915) overnight at 42°C, washed, and exposed to x-ray film. A quail glyceraldehyde-3-phosphate dehydrogenase (g3pdh) gene fragment (696–968 bp, 97.4% identical to chick) was used as an internal control. Message sizes were estimated by comparisons with RNA molecular weight markers (Invitrogen) run in the adjacent lane.

### *In vitro *transcription assay

Primers were designed to amplify each promoter region of interest. Constructs 3B-2256 (4201–6456 bp of *cIRK1 *5'-flanking region, AF375660), 3B-1229 (5228–6456 bp), 3B-531 (5926–6456 bp), and 3B-226 (6231–6456 bp) were generated by PCR, cloned into pBS-KS, and then subcloned into the multiple cloning site (between KpnI and XhoI) of pGL-3B (Promega). Constructs 3B-1089 (5368–6456 bp) and 3B-840 (5617–6456 bp) were obtained from a PCR fragment (1229 bp) with the 5'-region digested by SpeI and PvuII or XbaI, respectively. 3B-399 (6058–6456 bp) was derived from 3B-531 by removing the 5'-region by SacI and SmaI digestion, and 3B-206 was generated by exonuclease III digestion of 3B-226 followed by recircularization. 3B-109 (6348–6456 bp) was generated by SpeI and NheI digestion of 3B-206. Construction of 3B-206 with a mutated putative TATA box (3B-206dT) was accomplished by replacing the putative TATA box (TATTAA) with a StuI site (AGGCCT) by amplifying the original construct using primer sets designed to be complementary to sequences just outside the elements of interest, yet including StuI sites at their 5'-ends.

The chick fibroblast cell line DF1 was purchased from ATCC and cultured at 5% CO_2 _and 39°C in Dulbecco's minimum essential medium (DMEM) with 1.5 g/L sodium bicarbonate, 10% fetal calf serum, and 100 U/ml penicillin/streptomycin. The quail myoblast cell line Qm7 [25] was cultured in medium 199 with 10% tryptose phosphate, 10% fetal calf serum and 100 U/ml penicillin/streptomycin in 5% CO_2 _at 37°C. The ability of Qm7 cells to differentiate into myotube-like morphology was confirmed periodically as previously described [33]. One microgram of each construct with 0.1 μg of control vector pRL-TK were mixed in 50 μl of calcium phosphate buffer (140 mM NaCl, 5 mM KCl, 750 μM Na_2_HPO_4_, 6 mM dextrose, 25 mM HEPES at pH = 7.15, and 120 mM of CaCl_2_), and were incubated with 2 × 10^4 ^cells in 24-well dishes for 8 hrs in 500 μl of DMEM. The transfection mixes were replaced with fresh growth media and incubation was continued for 48 hrs. *In vitro *promoter activities were determined using the Dual-Luciferase Reporter Assay System (Promega). Briefly, transfected cells in each well were lysed in 100 μl of 1x Passive Lysis Buffer for 15 min at room temperature. Lysates were centrifuged briefly, and 20 μl of the supernatant was mixed with 100 μl of Luciferase Assay Reagent II, followed by 100 μl of SG reagent. Luciferase activities reflecting *cIRK1 *promoter activities and control thymidine kinase promoter activities were measured for 10 seconds after premeasurement periods of 2 seconds. All experiments were performed in duplicate and repeated at least 3 times (n= 3–6). The data were analyzed using a t-test assuming equal variance between two samples. Results are shown as fold increases ± standard error when compared with the basal luciferase activities from cells transfected with mock vector pGL-3B.

## Authors' contributions

HM obtained the sequence of cIRK1 genomic DNA and did all the database searches to identify motifs and control regions in the 5'-flanking region of *cIRK1*. He also conducted 5'RACE, Northern blot, cell culture, construction of deletion mutant, *in vitro *transcription analysis, and drafted the manuscript. LCK mapped much of the genomic structure and determined some of the sequence. EL and NK participated in maintenance of the cell lines and *in vitro *transcription analysis. JCO participated in design and coordination of the study and finalized the manuscript.

## References

[B1] Stanfield PR, Nakajima S, Nakajima Y (2002). Constitutively active and G-protein coupled inward rectifier K^+ ^channels: Kir2.0 and Kir3.0. Rev Physiol Biochem Pharmacol.

[B2] Zaritsky JJ, Eckman DM, Wellman GC, Nelson MT, Schwarz TL (2000). Targeted disruption of Kir2.1 and Kir2.2 genes reveals the essential role of the inwardly rectifying K^+ ^current in K^+^-mediated vasodilation. Circ Res.

[B3] Plaster NM, Tawil R, Tristani-Firouzi M, Canun S, Bendahhou S, Tsunoda A, Donaldson MR, Iannaccone ST, Brunt E, Barohn R (2001). Mutations in Kir2.1 cause the developmental and episodic electrical phenotypes of Andersen's syndrome. Cell.

[B4] Karschin C, Dissmann E, Stuhmer W, Karschin A (1996). IRK(1–3) and GIRK(1–4) inwardly rectifying K^+ ^channel mRNAs are differentially expressed in the adult rat brain. J Neurosci.

[B5] Kubo Y, Baldwin TJ, Jan YN, Jan LY (1993). Primary structure and functional expression of a mouse inward rectifier potassium channel. Nature.

[B6] Mi H, Deerinck TJ, Jones M, Ellisman MH, Schwarz TL (1996). Inwardly rectifying K^+ ^channels that may participate in K^+ ^buffering are localized in microvilli of Schwann cells. J Neurosci.

[B7] Morishige K, Takahashi N, Findlay I, Koyama H, Zanelli JS, Peterson C, Jenkins NA, Copeland NG, Mori N, Kurachi Y (1993). Molecular cloning, functional expression and localization of an inward rectifier potassium channel in the mouse brain. FEBS Lett.

[B8] Navaratnam DS, Escobar L, Covarrubias M, Oberholtzer JC (1995). Permeation properties and differential expression across the auditory receptor epithelium of an inward rectifier K^+ ^channel cloned from the chick inner ear. J Biol Chem.

[B9] Marcotti W, Geleoc GS, Lennan GW, Kros CJ (1999). Transient expression of an inwardly rectifying potassium conductance in developing inner and outer hair cells along the mouse cochlea. Pflugers Arch.

[B10] Hibino H, Horio Y, Inanobe A, Doi K, Ito M, Yamada M, Gotow T, Uchiyama Y, Kawamura M, Kubo T (1997). An ATP-dependent inwardly rectifying potassium channel, KAB-2 (Kir4. 1), in cochlear stria vascularis of inner ear: its specific subcellular localization and correlation with the formation of endocochlear potential. J Neurosci.

[B11] Redell JB, Tempel BL (1998). Multiple promoter elements interact to control the transcription of the potassium channel gene, KCNJ2. J Biol Chem.

[B12] Maruyama K, Sugano S (1994). Oligo-capping: a simple method to replace the cap structure of eukaryotic mRNAs with oligoribonucleotides. Gene.

[B13] Mantovani R (1998). A survey of 178 NF-Y binding CCAAT boxes. Nucleic Acids Res.

[B14] Wobbe CR, Struhl K (1990). Yeast and human TATA-binding proteins have nearly identical DNA sequence requirements for transcription in vitro. Mol Cell Biol.

[B15] Schoots O, Voskoglou T, Van Tol HH (1997). Genomic organization and promoter analysis of the human G-protein-coupled K^+ ^channel Kir3.1 (KCNJ3/HGIRK1). Genomics.

[B16] Spauschus A, Lentes KU, Wischmeyer E, Dissmann E, Karschin C, Karschin A (1996). A G-protein-activated inwardly rectifying K^+ ^channel (GIRK4) from human hippocampus associates with other GIRK channels. J Neurosci.

[B17] Wymore RS, Negulescu D, Kinoshita K, Kalman K, Aiyar J, Gutman GA, Chandy KG (1996). Characterization of the transcription unit of mouse Kv1.4, a voltage-gated potassium channel gene. J Biol Chem.

[B18] Mori Y, Folco E, Koren G (1995). GH3 cell-specific expression of Kv1.5 gene. Regulation by a silencer containing a dinucleotide repetitive element. J Biol Chem.

[B19] Gan L, Hahn SJ, Kaczmarek LK (1999). Cell type-specific expression of the Kv3.1 gene is mediated by a negative element in the 5' untranslated region of the Kv3.1 promoter. J Neurochem.

[B20] Gan L, Perney TM, Kaczmarek LK (1996). Cloning and characterization of the promoter for a potassium channel expressed in high frequency firing neurons. J Biol Chem.

[B21] Kyo S, Takakura M, Taira T, Kanaya T, Itoh H, Yutsudo M, Ariga H, Inoue M (2000). Sp1 cooperates with c-Myc to activate transcription of the human telomerase reverse transcriptase gene (hTERT). Nucleic Acids Res.

[B22] Blondeau N, Widmann C, Lazdunski M, Heurteaux C (2001). Activation of the nuclear factor-kappaB is a key event in brain tolerance. J Neurosci.

[B23] Mattson MP, Culmsee C, Yu Z, Camandola S (2000). Roles of nuclear factor kappaB in neuronal survival and plasticity. J Neurochem.

[B24] Akazawa C, Ishibashi M, Shimizu C, Nakanishi S, Kageyama R (1995). A mammalian helix-loop-helix factor structurally related to the product of Drosophila proneural gene atonal is a positive transcriptional regulator expressed in the developing nervous system. J Biol Chem.

[B25] Pinney DF, de la Brousse FC, Faerman A, Shani M, Maruyama K, Emerson CP (1995). Quail myoD is regulated by a complex array of cis-acting control sequences. Dev Biol.

[B26] Ben-Arie N, McCall AE, Berkman S, Eichele G, Bellen HJ, Zoghbi HY (1996). Evolutionary conservation of sequence and expression of the bHLH protein Atonal suggests a conserved role in neurogenesis. Hum Mol Genet.

[B27] Rusch A, Erway LC, Oliver D, Vennstrom B, Forrest D (1998). Thyroid hormone receptor beta-dependent expression of a potassium conductance in inner hair cells at the onset of hearing. Proc Natl Acad Sci U S A.

[B28] Xu D, Popov N, Hou M, Wang Q, Bjorkholm M, Gruber A, Menkel AR, Henriksson M (2001). Switch from Myc/Max to Mad1/Max binding and decrease in histone acetylation at the telomerase reverse transcriptase promoter during differentiation of HL60 cells. Proc Natl Acad Sci U S A.

[B29] Jethanandani P, Goldberg E (2001). ldhc expression in non-germ cell nuclei is repressed by NF-I binding. J Biol Chem.

[B30] Affolter M, Percival-Smith A, Muller M, Leupin W, Gehring WJ (1990). DNA binding properties of the purified Antennapedia homeodomain. Proc Natl Acad Sci U S A.

[B31] Jaenisch R, Bird A (2003). Epigenetic regulation of gene expression: how the genome integrates intrinsic and environmental signals. Nat Genet.

[B32] Wingender E, Chen X, Hehl R, Karas H, Liebich I, Matys V, Meinhardt T, Pruss M, Reuter I, Schacherer F (2000). TRANSFAC: an integrated system for gene expression regulation. Nucleic Acids Research.

[B33] Antin PB, Ordahl CP (1991). Isolation and characterization of an avian myogenic cell line. Dev Biol.

